# Medical Society of the County of Niagara

**Published:** 1906-05

**Authors:** John A. Rafter


### Medical Society of the County of Genesee

Reported By JOHN A. RAFTER, M. D.

### Medical Society of the County of Niagara

Reported By JOHN A. RAFTER, M. D.

The regular meeting of the Medical Society of the County of Niagara was held at Lockport, Tuesday evening, April 3, at 8.30 P. M. Dr. A. N. Moore, president; Dr. Frank A. Crosby, secretary. Members present were Drs. Baker, Palmer, Preisch, Ringueberg, Crosby, Moore, Kittinger, Mayne, McNamara, and Cole, of Lockport; Drs. Guillemont, Potter, Chapin, Miller, and Scott of Niagara Falls; Dr. Chas. W. Clendennan of North Tonawanda; Dr. Shoemaker of Newfane, and Dr. Ernst of Gasport. After the routine business of the Society was concluded, Dr. De Lancey Rochester of Buffalo, presented a paper on "The Hygienic Managment of Chronic Nephritis." The paper was well received and a vote of thanks tendered to Dr. Rochester for his interesting thesis.

Dr. Miller of Niagara Falls, and Dr. Baker of Lockport, were elected delegates to the State Medical Society, and Dr.

Charles N. Palmer was elected delegate to represent the County Society at the eighth district branch of the State Medical Society. The next regular meeting will be held at the Hotel Sheldon, North Tonawanda, Tuesday, May 1, 1906.

---

